# Exploring ascomycete diversity in Yunnan, China I: resolving ambiguous taxa in *Phaeothecoidiellaceae* and investigating conservation implications of fungi

**DOI:** 10.3389/fcimb.2023.1252387

**Published:** 2023-09-04

**Authors:** Sinang Hongsanan, Rungtiwa Phookamsak, Darbhe Jayarama Bhat, Dhanushka N. Wanasinghe, Itthayakorn Promputtha, Nakarin Suwannarach, Diana Sandamali, Saisamorn Lumyong, Jianchu Xu, Ning Xie

**Affiliations:** ^1^ Center of Excellence in Microbial Diversity and Sustainable Utilization, Chiang Mai University, Chiang Mai, Thailand; ^2^ Shenzhen Key Laboratory of Microbial Genetic Engineering, College of Life Sciences and Oceanography, Shenzhen University, Shenzhen, China; ^3^ Honghe Center for Mountain Futures, Kunming Institute of Botany, Chinese Academy of Sciences, Honghe, Yunnan, China; ^4^ CIFOR-ICRAF China Country Program, Kunming, Yunnan, China; ^5^ Centre for Mountain Futures (CMF), Kunming Institute of Botany, Kunming, Yunnan, China; ^6^ Department of Botany and Microbiology, College of Science, King Saud University, Riyadh, Saudi Arabia; ^7^ Vishnugupta Vishwavidyapeetam, Gokarna, India; ^8^ Department of Biology, Faculty of Science, Chiang Mai University, Chiang Mai, Thailand; ^9^ Center of Excellence in Fungal Research, Mae Fah Luang University, Chiang Rai, Thailand; ^10^ Academy of Science, The Royal Society of Thailand, Bangkok, Thailand

**Keywords:** *Dothideomycetes*, fungal taxonomy, *Mycosphaerellales*, novel taxa, sootyblotch/flyspeck fungi

## Abstract

Yunnan, located in southwestern China, is known for its high fungal diversity, and many of which are endemic to the region. As part of our ongoing studies on fungi in Yunnan, we introduce two new genera in *Phaeothecoidiellaceae* (*Mycosphaerellales*), to accommodate one *Repetophragma*-like and another *Stomiopeltis*-like taxa. *Pseudorepetophragma* gen. nov. is introduced herein as a monotypic genus to accommodate *P. zygopetali* comb. nov.(≡ *Repetophragma zygopetali*), whereas *Pseudostomiopeltis* gen. nov. is introduced to accommodate *Ps. xishuangbannaensis* gen. et sp. nov. and *Ps. phyllanthi* comb. nov.(≡ *Stomiopeltis phyllanthi*), based on a new collection from Yunnan. In addition, *Stomiopeltis sinensis* is transferred to *Exopassalora* as *E. sinensis* comb. nov. due to its phylogenetic affinity and grouped with *E. zambiae*, the generic type of *Exopassalora*. This study provides new insights into the biodiversity of fungal species in this region and adds to our understanding of their ecological roles, as well as the resolution to ambiguous taxa in *Phaeothecoidiellaceae*.

## 1 Introduction

China is home to diverse climates and environments containing four of the world’s 36 biodiversity hotspots; of which, three hotspots, the mountain ranges of Southwest China, Eastern Himalaya, and Indo-Burma, intersect with the Yunnan Province (Feng and Yang, 2018; Cai et al., 2019). Yunnan has diverse climate types and environments and is significantly affected by the monsoon rains (abundant rainfall and resultant humid tropical evergreen rainforests). Diverse environments, complex topography and geography, and highly variable plant species allow fungi to specialize and flourish, and these also affect the fungal growth and distribution (Yang et al., 2004; Zhu et al., 2006; Feng and Yang, 2018; Wanasinghe et al., 2020). Furthermore, Yunnan is an agricultural province that cultivates a wide variety of agricultural and horticultural crops such as coffee (Arabica), commercial flowers, fruits (e.g., grapes, passion fruits, bananas, and mangoes), grains (e.g., rice), rubber, sugarcane, tea (Pu’er), tobacco, plantation trees (e.g., Yunnan pine, bamboo, and teak), vegetables as well as wild edible mushrooms (Li et al., 2011; Frayer et al., 2014; Zhang et al., 2014; Shen et al., 2017; ORO Yunnan Commerce, 2023). This also led Yunnan to host a high diversity of fungi that has not been studied so far.

According to Feng and Yang (2018), there may be approximately 104,000 fungal species existing in Yunnan, but only 6,000 are described, including roughly 3,000 species of higher fungi (*Ascomycota* and *Basidiomycota*), indicating that fewer than 5% of known species have been described in this province. Two prominent regions of Yunnan have been well documented on fungal diversity, *viz*., the Eastern Himalayas and Hengduan Mountains in northwestern Yunnan and the tropical region in southern and southwestern Yunnan, while the other parts remain a huge challenge (Feng and Yang, 2018).

*Dothideomycetes* is the largest and most diverse class of *Ascomycota*, containing 49 orders, 223 families, 1,765 genera, and over 19,000 species (Hongsanan et al., 2020a; Hongsanan et al., 2020b; Wijayawardene et al., 2022). *Dothideomycetes* was previously known as *Loculoascomycetes* (Nannfeldt, 1932; Luttrell, 1955; Barr, 1979; Eriksson, 1981; Barr and Huhndorf, 2001). The taxa in this class are commonly characterized by bitunicate, fissitunicate asci that are also shared with taxa in *Arthoniomycetes* and *Eurotiomycetes* (Hyde et al., 2013; Hongsanan et al., 2020a; Hongsanan et al., 2020b; Pem et al., 2021). However, *Dothideomycetes* can be distinguished from these two classes based on multigene phylogeny, their evolutionary relationships from molecular clock analysis, and ecological niches (Geiser et al., 2015; Schoch and Grube, 2015; Liu et al., 2017; Hongsanan et al., 2020a; Hongsanan et al., 2020b; Wijayawardene et al., 2022). Even though several genera and families of *Dothideomycetes* have been well-resolved based on molecular phylogeny in the past two decades, there are still over 200 genera that could not be classified into any families or orders due to the lack of informative phylogenetic markers and uncertain morphological features (Thambugala et al., 2014; Phillips et al., 2019; Abdollahzadeh et al., 2020; Haridas et al., 2020; Hongsanan et al., 2020a; Hongsanan et al., 2020b; Pem et al., 2021; Wijayawardene et al., 2022).

One of the *Dothideomycetes* orders, *Mycosphaerellales*, comprises eight families, 230 genera (including the doubtful taxa of *Mycosphaerellaceae* and genera *incertae sedis*), and over 4,200 species (Abdollahzadeh et al., 2020; Wijayawardene et al., 2022). The order encompassed ecologically and morphologically diverse fungi, including endophytes, epiphytes, lichenicolous fungi, phytopathogens, and saprobes (Abdollahzadeh et al., 2020). Of these, many genera of *Mycosphaerellaceae* are well-known as important plant pathogens that are quarantine regulated, such as *Pseudocercospora angolensis* causing fruit and leaf spot disease on citrus, and *Septoria malagutii* causing angular leaf spot on potato (Quaedvlieg et al., 2012; Videira et al., 2017). *Mycosphaerellales* was introduced by Kirk et al. (2001) to accommodate *Mycosphaerellaceae* and is characterized by small perithecial ascomata, often immersed to erumpent through the host surface by black papilla with lysigenous ostiole; the peridium composed of thin pseudoparenchymatous cells; lacking interascal tissue; asci fissitunicate, ovoid to saccate; and ascospores hyaline, septate, lacking mucilaginous sheath. Subsequently, Schoch et al. (2006) and Kirk et al. (2008) placed *Mycosphaerellaceae* in *Capnodiales*, and hence *Mycosphaerellales* was treated as a synonym of *Capnodiales*. Phylogenetic analyses of a concatenated large subunit (LSU), *tef1-α*, and *rpb2* sequence dataset conducted by Abdollahzadeh et al. (2020) revealed that *Mycosphaerellales* represents a robust clade, accommodating eight families, viz., *Cystocoleaceae*, *Dissoconiaceae*, *Extremaceae*, *Mycosphaerellaceae*, *Neodevriesiaceae*, *Phaeothecoidiellaceae*, *Schizothyriaceae*, and *Teratosphaeriaceae*. Hence, Abdollahzadeh et al. (2020) resurrected the order *Mycosphaerellales* as distinct from *Capnodiales* and also provided an amended description for the order.

Family *Phaeothecoidiellaceae*, a member of *Mycosphaerellales*, was introduced by Hongsanan et al. (2017) to accommodate the genera *Chaetothyrina*, *Houjia*, and *Phaeothecoidiella*. Members of this family are well-known as sooty blotch/flyspeck fungi which were frequently found as epiphytes or pathogens on fruits, leaves, and stems (Hongsanan et al., 2017; Hongsanan et al., 2020b). Subsequently, Zeng et al. (2018) introduced a new genus *Translucidithyrium* to this family. Hongsanan et al. (2020b) updated the taxonomic status of *Phaeothecoidiellaceae* and treated *Nowamycetaceae* as a synonym of *Phaeothecoidiellaceae* by transferring *Nowamyces* to *Phaeothecoidiellaceae*. Therefore, Hongsanan et al. (2020b) accepted five genera in *Phaeothecoidiellaceae*, while Wijayawardene et al. (2022) listed nine genera in this family, viz., *Chaetothyrina*, *Exopassalora*, *Houjia*, *Neochaetothyrina*, *Nowamyces*, *Phaeothecoidiella*, *Rivilata*, *Sporidesmajora*, and *Translucidithyrium*.

*Exopassalora* was established by Videira et al. (2017) to accommodate a single species, previously described as *Passalora zambiae* (Crous et al., 2004). The genus is characterized by irregularly branched, septate mycelium, composed of brown hyphae, with dark brown chlamydospore-like hyphal swellings; medium brown, smooth, simple or branched conidiophore-bearing terminal and intercalary, subcylindrical, pale to medium brown sympodially proliferating, polyblastic, conidiogenous cells, with darkened, conspicuous conidiogenous loci, and medium brown, smooth, narrowly ellipsoidal, tapering to subtruncate conidia with thickened and darkened hila and produced in simple or branched chains (Videira et al., 2017). According to Videira et al. (2017), *Exopassalora zambiae* formed a clade with *Exopassalora* sp. CBS 118964 in *Phaeothecoidiellaceae* and was phylogenetically distant from other *Passalora* species in *Mycosphaerellaceae*. Videira et al. (2017) reassigned most *Passalora sensu lata* into new genera.

*Repetophragma*, typified by *R. biseptatum* (*≡Sporidesmium biseptatum*), was introduced by Subramanian (1992), to accommodate *Sporidesmium*-like species with holoblastic, annellidic, percurrently proliferating conidiogenous cells and phragmoseptate conidia and initially included nine species previously known in *Sporidesmium*. Subsequently, many species were accommodated in *Repetophragma* based solely on morphological characterizations (McKenzie, 1995; Mena-Portales et al., 2000; Wu and Zhuang, 2005; Castañeda-Ruiz et al., 2006; Marincowitz et al., 2008; Silvera-Simón et al., 2009; Castañeda-Ruiz et al., 2011; Rambelli et al., 2011; Castañeda-Ruiz et al., 2013; Ma et al., 2014; Buyck et al., 2017; Wang et al., 2017; Ai et al., 2019). Even though, there are 40 species epithets available for *Repetophragma* in Index Fungorum (2023), most of them lack molecular data to clarify their phylogenetic placements. Only four species, namely, *R. goidanichii, R. inflatum, R. ontariense,* and *R. zygopetali*, have molecular data available in GenBank. Of these, *R. ontariense* was treated as a synonym of *Vargamyces aquaticus* (*Amniculicolaceae, Pleosporales*) by Hernández-Restrepo et al. (2017), whereas *R. goidanichii* was placed in *Venturiaceae* (*Venturiales, Dothideomycetes*) and *R. inflatum* was placed in *Xylariales incertae sedis* (Hernández-Restrepo et al., 2017). This concurs with the phylogenetic studies conducted by Shenoy et al. (2006). Unfortunately, the genus has not yet been clarified in terms of its generic placement based on the molecular data of the type species, *R. biseptatum*. Hence, the phylogenetic affinity of *Repetophragma sensu stricto* remains uncertain pending further study.

*Stomiopeltis* was introduced by Theissen (1914) to initially accommodate a single species *S. aspersa* which was collected on the leaves of *Laurus* sp. in India. Subsequently, many species—mostly in the 19th century—that lacked molecular data to clarify their phylogenetic placement were included in the genus (Luttrell, 1946; Müller and von Arx, 1962; Index Fungorum, 2023). *Stomiopeltis* has been reported as a pathogen causing sooty blotch/flyspeck disease but has also been found as a saprobe on fruits (Mayfield et al., 2013; Ajitomi et al., 2017; Jayasiri et al., 2019; Batzer et al., 2022). The genus is scarcely known, and only a few species have molecular data available in GenBank (Crous et al., 2019; Jayasiri et al., 2019; Renard et al., 2020). Most *Stomiopeltis*-like isolates were treated as *Stomiopeltis* sp. (Ajitomi et al., 2017; Batzer et al., 2022). An updated taxonomic treatment of *Stomiopeltis* was carried out by Zeng et al. (2019) who treated the genus in *Capnodiales incertae sedis*, and this was followed by Hongsanan et al. (2020b) and Wijayawardene et al. (2022). Whereas Renard et al. (2020) demonstrated that *Stomiopeltis* is polyphyletic, forming clades within the orders *Microthyriales* and *Venturiales*. Since the type species of *Stomiopeltis*, *S. aspersa*, has not yet been sequenced, the phylogenetic status of *Stomiopeltis* remains doubtful and also pending further study.

Several taxonomic studies of *Dothideomycetes* in Yunnan have been published in the past few years (Tibpromma et al., 2018; Phookamsak et al., 2019; Dong et al., 2020; Hyde et al., 2020; Li et al., 2020; Mortimer et al., 2021; Wanasinghe et al., 2021; Yang et al., 2022). Even though these studies resulted in a substantial increase in the number of described microfungi in Yunnan, there is still a glaring knowledge gap in our understanding of the fungi in this region. The present study aims to introduce two novel genera, one novel species, and three new combinations of *Phaeothecoidiellaceae* in Yunnan, based on molecular phylogeny coupled with morphological characteristics.

## 2 Materials and methods

### 2.1 Sample collection, morphological examination, isolation, and preservation

The sample was collected from Xishuangbanna, Yunnan Province, China in 2021. The sample was stored in a plastic Ziploc bag and returned to the laboratory for observation and examination. Fungal fruiting bodies on host substrates were observed using an Olympus SZ61 series stereomicroscope. Micro-morphologies on squash-mount slides were observed and photographed using a Nikon ECLIPSE Ni-U compound microscope equipped with a Nikon DS-Ri2 camera. Congo red was used to stain the conidiomatal centrum for clarity of conidiophores and conidiogenous cells. Lacto-glycerol was added to preserve important morphological features on permanent slides and edges of the coverslip were sealed with nail polish. All morphological features were measured using Tarosoft (R) Image FrameWork version 0.9.7., and the photographic plate was processed using Adobe Photoshop CS6 software (Adobe Systems Inc., San Jose, CA, USA).

Fungal pure culture was obtained by single spore isolation, according to the methods described in Senanayake et al. (2020). The germinated spores were transferred to freshly sterilized potato dextrose agar (PDA) and incubated under normal light at 20°C–25°C. Culture characteristics were observed and recorded after one- and four-week intervals. The specimen was deposited in the Herbarium of Cryptogams Kunming Institute of Botany Academia Sinica (KUN-HKAS), China. Axenic living cultures were preserved at the collection of Rungtiwa Phookamsak housed at Honghe Center for Mountain Futures (RPC) and duplicated in the Culture Collection of the Herbarium of Cryptogams Kunming Institute of Botany, Academia Sinica (KUNCC) Kunming, China. Index Fungorum numbers are provided for the newly described taxa.

### 2.2 DNA extraction, PCR amplification, and sequencing

Fungal genomic DNA was extracted from mycelia that grow on PDA for four weeks by using the Biospin Fungus Genomic DNA Extraction Kit (BioFlux^®^, Hangzhou, China) according to the manufacturer’s protocol. The conditions for the polymerase chain reaction (PCR) were determined using the primer pairs LR0R/LR5 (Vilgalys and Hester, 1990) to amplify the 28S large subunit region (LSU), and ITS4/ITS5 (White et al., 1990) to amplify the internal transcribed spacer region (ITS: ITS1-5.8S-ITS2). The amplification of ITS and LSU was carried out with setting times and temperatures for the initialization, denaturation, annealing, and final extension periods following Phookamsak et al. (2017).

The final PCR reaction component was 25 µl, containing 12.5 μl Master Mix (mixture of *EasyTaq*TM DNA Polymerase, dNTPs, and optimized buffer; Beijing TransGen Biotech Co., Ltd., Chaoyang District, Beijing, China), 8.5 µl of double-distilled water (ddH_2_O), 1 μl of each forward and reverse primer (10 μm), and 2 μl DNA template. The PCR products were sent to TsingKe Biological Technology, Kunming City, Yunnan Province, China, for purification and Sanger sequencing. The consensus sequences of the newly generated strains are available to the scientific community via submission to GenBank.

### 2.3 Sequence alignment and phylogenetic analyses

The chromatograms of sequence results were checked, manually edited, trimmed, and assembled into consensus sequences using SeqMan Pro version 11.1.0 (DNASTAR, Inc. Madison, WI, USA). The consensus sequences of the newly generated strains were blasted using the nucleotide BLAST search tool on the NCBI website to search for closely related species in the GenBank database. Sequence data obtained from this study, the closely related species from nucleotide BLAST search, and previous studies were downloaded from GenBank to supplement the datasets (**Table 1**). The dataset was prepared to generate the phylogenetic trees for taxa in *Mycosphaerellales*. Each sequence dataset was aligned using MAFFT (Katoh et al., 2019) and checked manually in Bioedit (Hall, 2004). Maximum likelihood (ML) and Bayesian analysis (BI) were conducted based on the individual datasets.

**Table 1:** Species details and GenBank accession numbers used in the phylogenetic analysis of *Phaeothecoidiellaceae* (*Mycosphaerellales*) and other related families and orders.

Species	Strain no.	ITS	LSU
***Botryosphaeria fusispora***	**MFLUCC 10-0098**	**JX646789**	**JX646806**
***Capnodium coartatum***	**MFLUCC 10-0069**	–	**NG_058834**
*Capnodium gardeniorum*	CPC 14327	–	GU301807
***Chaetothyrina guttulata***	**MFLUCC 15-1081**	**NR_153923**	**NG_058932**
*Chaetothyrina musarum*	MFLUCC 15-0383	KX372275	KU710171
***Chaetothyriothecium elegans***	**CPC 21375**	–	**KF268420**
***Cladosporium herbarum***	**CBS 121621/CPC 12177**	**MH863124**	**MH874676**
***Dissoconium aciculare***	**CBS 342.82**	**NR_119427**	**NG_059076**
***Exopassalora sinensis***	**MFLU 18-2203/C450**	**MK347799**	**MK348018**
***Exopassalora zambiae***	**CBS 112971**	**AY725523**	**DQ246264**
*Heliocephala elegans*	MUCL 39003	HQ333478	HQ333478
***Heliocephala zimbabweensis***	**MUCL 40019**	**HQ333481**	**HQ333481**
***Houjia pomigena***	**CBS 125224**	**NR_156364**	**NG_058486**
***Houjia yanglingensis***	**CBS 125225**	**MH863464**	**NG_064220**
***Lasiodiplodia gonubiensis***	**CBS 115812**	**AY639595**	**DQ377902**
*Microthyrium microscopicum*	CBS 115976	–	GU301846
***Microthyrium propagulensis***	**IFRD 9037**	–	**KU948989**
***Neochaetothyrina syzygii***	**CBS 147073**	**NR_173054**	**NG_076743**
***Nowamyces globulus***	**CBS 144598**	**NR_165606**	**NG_067915**
***Nowamyces piperitae***	**CBS 143490**	**NR_165607**	**NG_067916**
***Phaeothecoidiella illinoisensis***	**CBS 125223**	**MH863463**	**MH874963**
***Phaeothecoidiella missouriensis***	**CBS 125222**	**MH863462**	**MH874962**
***Polyphialoseptoria terminaliae***	**CBS 135106**	**NR_156559**	**KF251717**
***Pseudopenidiella piceae***	**CBS 131453**	**JX069868**	**JX069852**
***Pseudorepetophragma zygopali***	**VIC 42946**	**NG_060158**	**KT732418**
***Pseudostomiopeltis phyllanthi***	**MFLU 18-2115/C241**	**MK347734**	**MK347951**
***Pseudostomiopeltis xishuangbannaensis***	**Xh5/RPC 21-031**	**OR233596**	**OR233594**
***Pseudoveronaea ellipsoidea***	**CBS 132085**	**NR_111367**	**MH877464**
***Rachicladosporium luculiae***	**CBS 121620**	**NR_160222**	**MH874675**
***Ramularia eucalypti***	**CPC 13043/CBS 120726**	**NR_145121**	**KF251834**
***Ramularia pusilla***	**CBS 124973**	**NR_154917**	**NG_058152**
***Readerielliopsis fuscoporiae***	**CPC 24637/CBS:139900**	**KR476720**	**KR476755**
***Readeriellopsis guyanensis***	**CBS 117550**	**EU707900**	**FJ493211**
*Schizothyrium pomi*	Flyspeck1924-Zj001	AY598848	AY598894
*Schizothyrium* sp.	SP1 13 (5)	–	MN065462
*Schizothyrium* sp.	SP2-69	–	MN065460
***Schizothyrium wisconsinensis***	**MSTA8a/CBS 118950**	**AY598853**	**AY598897**
***Sporidesmajora pennsylvaniensis***	**CBS 125229**	**MF951287**	**MH874965**
*Stomiopeltis betulae*	CBS 114420	GU214701	GU214701
*Stomiopeltis* sp.	RS 5.2 NC1_18C1d	–	FJ147164
*Stomiopeltis* sp.	S1-2.G2 U1B.CCRS.8.20.96.1R=GP001	AY160162	–
*Stomiopeltis* sp.	CCRS4R-Gp002	AY598880	AY598919
*Stomiopeltis* sp.	S1-10.G3 M5A.MHCRS.9.11.96.5R	AY160168	–
*Stomiopeltis* sp.	S1-5.G4 M2B.Bertie.8.28.96.2R	AY160165	–
*Stomiopeltis* sp.	MHCRS11R-Gp010	AY598881	AY598920
*Stomiopeltis* sp.	It-s	LC190412	LC190414
*Stomiopeltis* sp.	To-f	LC190413	LC190415
*Stomiopeltis* sp.	RS3.3 SP8_291Ca/SP8-291	MN065477	MN065477
*Stomiopeltis* sp.	RS3.4 SP12_391Ca	MN065478	MN065478
*Stomiopeltis* sp.	RS3.1 SP13_438Fb/SP13-438	MN065476	MN065476
*Stomiopeltis* sp.	RS7 SP5_150Ca/SP5-158	MN065479	MN065479
*Stomiopeltis* sp.	S1-13.G3 M6.CHS#5	AY160170	–
*Stomiopeltis* sp.	RS4.1/T58C4d	JQ358787	JX042482
***Stomiopeltis syzygii***	**CPC 36323**	**NR_166321**	**NG_068323**
*Stomiopeltis versicolor*	GA3_23C2b/RS5.1	FJ438375	FJ147163
*Stomiopeltis*-like sp.	RS4.1 TN1_6.3E2a	–	FJ147162
*Stomiopeltis*-like sp.	RS1 PEC6a	–	AY598921
*Stomiopeltis*-like sp.	RS2.1 AHC3a	–	AY598922
*Stomiopeltis*-like sp.	RS3.2 KY4 11.2F2b	–	FJ147161
*Stomiopeltis*-like sp.	RS3.1 MI3_24F1a	–	FJ147160
*Stomiopeltis*-like sp.	RS7.2/T49A1c	–	JX042483
*Stomiopeltis*-like sp.	RS7.1/T36A1b	JQ358788	JX042481
*Toxicocladosporium irritans*	CBS 185.58	MH857749	MH869283
***Translucidithyrium chinense***	**IFRDCC 3000**	**NR_176736**	**NG_081480**
***Translucidithyrium thailandicum***	**MFLUCC 16-0362**	**NR_161061**	**MG993048**
***Uwebraunia dekkeri***	**STE-U 1535/CBS 567.89**	**NR_119428**	**EU019268**

The newly generated sequences are indicated in red, and the ex-type strains are in bold. The missing sequences are indicated by “–”.

Maximum likelihood analyses were performed in RAxML-HPC v.8 on the XSEDE (8.2.12) tool in the online web portal CIPRES Science Gateway v. 3.3 using default settings but following adjustments with 1,000 bootstrap replications. The BI analyses were conducted via the same web portal as in ML, with two different runs, and six chains were executed. The initial 25% of sample trees were treated as burn-in and discarded. The trees were visualized using Figtree v. 1.4.0 (Rambaut, 2014) and edited in Adobe Illustrator version 20.0.0.

## 3 Results

### 3.1 Molecular phylogeny

**Figure 1:**
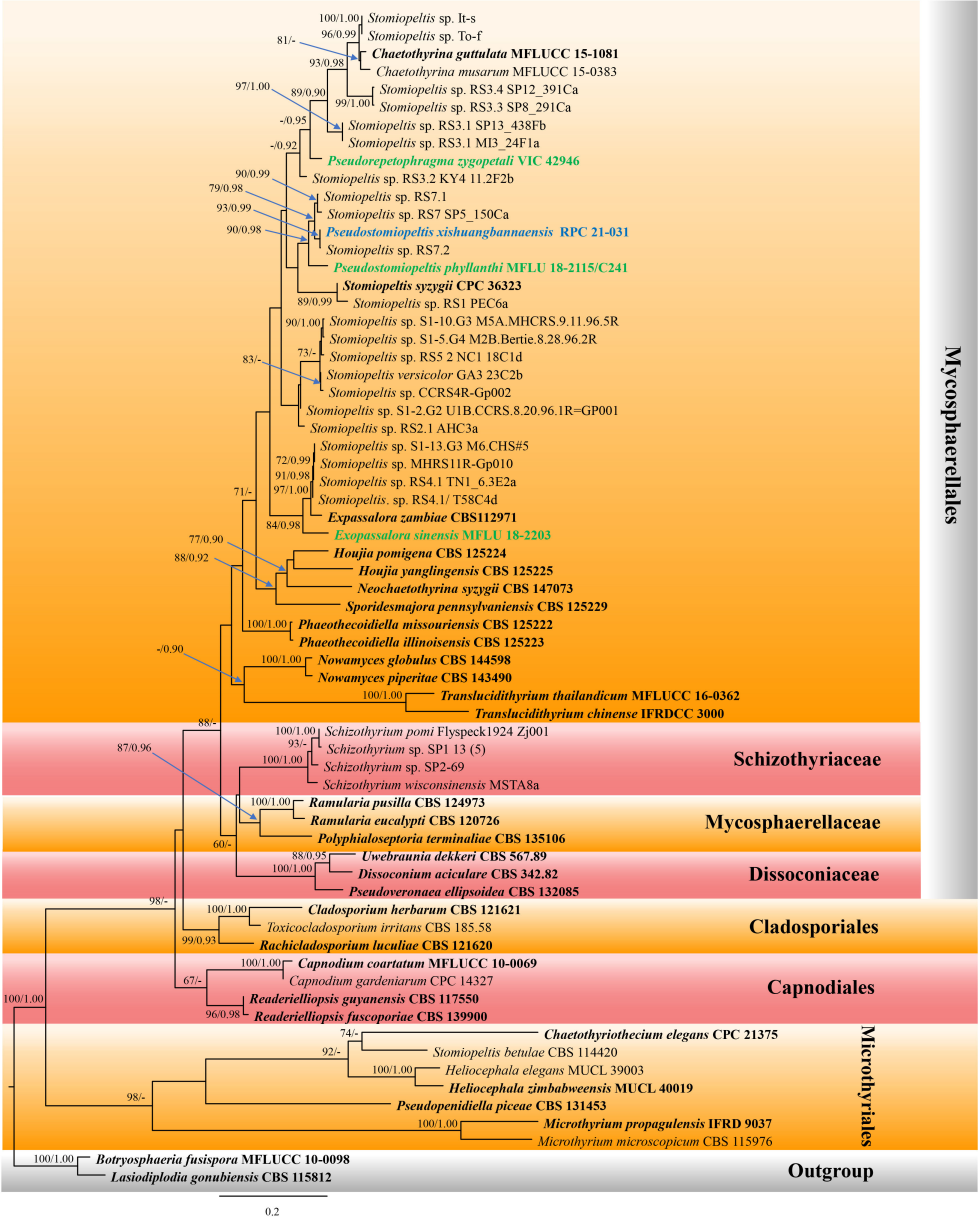
Phylogram of the best-scoring maximum likelihood (ML) consensus tree based on a combined dataset (ITS and LSU) of *Pseudostomiopeltis*. The new species is indicated in blue, and the new combination species are indicated in green. Isolates from type materials are in bold. The ML ultrafast bootstrap and Bayesian PP values greater than 70% and 0.95 are shown at the nodes. The tree is rooted with *Botryosphaeria fusispora* (MFLUCC 10-0098) and *Lasiodiplodia gonubiensis* (CBS 115812).

The phylogenetic tree which represented novel taxa in *Phaeothecoidiellaceae* was constructed using sequence data from ITS and LSU genes. A total of 66 strains of taxa in the family *Phaeothecoidiellaceae*, representative of other related families in *Capnodiales*, *Cladosporiales*, *Microthyriales*, and *Mycosphaerellales*, were included, with two strains of *Botryosphaeria fusispora* (MFLUCC 10-0098) and *Lasiodiplodia gonubiensis* (CBS 115812) as the outgroup. The aligned dataset contained 1,808 characters, including gaps. The best-scoring RAxML tree was selected to represent the relationships among taxa, with a final likelihood value of -17,660.527024. The matrix contained 1,121 distinct alignment patterns, with estimated base frequencies of A = 0.240165, C = 0.248028, G = 0.296115, T = 0.215692; substitution rates AC = 1.209829, AG = 1.862907, AT = 1.368661, CG = 1.131373, CT = 4.503418, GT = 1.000000; and gamma distribution shape parameter α = 0.495210 (**Figure 1**). For BI analysis, GTR + I + G was selected as the best-fit model by AIC in MrModeltest for each gene (ITS and LSU). Six simultaneous Markov chains were run for 3,000,000 generations, and trees were sampled every 100 generations. The first 25% of trees were discarded as the burn-in phase of the analyses, and the remaining trees were used for calculating posterior probabilities in the majority rule consensus tree (the critical value for the topological convergence diagnostic is 0.01), of which the final average standard deviation of split frequencies at the end of total MCMC generations was 0.009273.

Our new isolate shared the branch length with *Stomiopeltis* sp. (RS7.2), with significant support (93% ML, 0.99 BYPP, **Figure 1**), and formed a well-resolved subclade with *Stomiopeltis* sp. (strains RS7 SP5_150Ca and RS7.1) within *Phaeothecoidiellaceae* (*Mycosphaerellales*). According to Renard et al. (2020), *Stomiopeltis* is polyphyletic. Moreover, in comparison with the type of *Stomiopeltis* examined by Zeng et al. (2018), our new isolate is morphologically distinguishable from *S. aspersa*, the generic type of *Stomiopeltis*, based on the upper wall arrangement. Hence, the new genus *Pseudostomiopeltis* is introduced herein to accommodate the new species, *Ps. xishuangbannaensis*, and *Stomiopeltis sensu lato* in this subclade. Furthermore, *Repetophragma zygopetali* (VIC 42946, ex-type strain) formed an independent lineage basal to the genus *Chaetothyrina* and *Stomiopeltis* spp. (strains It-s, To-f, RS3.3 SP8_291Ca, RS3.4 SP12_391Ca, RS3.1 SP13_438Fb, and RS3.1 MI3_24F1a). According to Shenoy et al. (2006), *Repetophragma* has shown to be polyphyletic, affiliating in different families and orders of *Dothideomycetes* and *Sordariomycetes*. Moreover, the generic type of *Repetophragma*, *R. biseptatum*, lacks molecular data to clarify its phylogenetic placement. Hence, the new genus *Pseudorepetophragma* is introduced herein to accommodate *R. zygopetali* as *Pseudorepetophragma zygopetali* comb. nov. based on phylogenetic evidence and morphological distinction in the conidiogenous proliferations, whereas *Stomiopeltis sinensis* clustered with *Exopassalora zambiae* (CBS 125225, ex-type strain) and *Stomiopeltis* spp. (strains S1-13.G3 M6.CHS#5, MHCRS11R-Gp010, RS4.1 TN1_6.3E2a, and RS4.1/T58C4d) with significant support (84% ML, 0.98 BYPP; **Figure 1**). Thus, *S. sinensis* is also transferred to *Exopassalora* as *E. sinensis* comb. nov.

### 3.2 Taxonomy

***Exopassalora sinensis*** (Jayasiri, E.B.G. Jones and K.D. Hyde) Phookamsak and Hongsanan, comb. nov.

*Index Fungorum number*: IF 900623

≡ *Stomiopeltis sinensis* Jayasiri, E.B.G. Jones and K.D. Hyde, in Jayasiri, Hyde, Jones, McKenzie, Jeewon, Phillips, Bhat, Wanasinghe, Liu, Lu, Kang, Xu and Karunarathna, Mycosphere 10(1): 129 (2019)

*Detailed description*: See Jayasiri et al. (2019).

*Notes*: *Exopassalora sinensis* was first introduced as a saprobic fungus on decaying fruit pericarp of *Harpephyllum* in Yunnan, China (Jayasiri et al., 2019). The species is represented by its sexual morph having superficial, rounded thyriothecia, dark brown, *textura angularis* cell-layered peridium, 4-spored, fissitunicate, oblong to subglobose asci embedded in filiform, unbranched, septate pseudoparaphyses, and hyaline, obovoid to ellipsoid, 1-septate ascospores (Jayasiri et al., 2019). Phylogenetic analyses (**Figure 1**) demonstrated that the species formed a well-resolved clade with *Exopassalora zambiae* (CBS 125225, ex-type strain) and unnamed *Stomiopeltis* spp. within *Phaeothecoidiellaceae*. Morphologically, the species could not be compared with *E. zambiae* due to the representative different morphs. Therefore, the species is transferred to *Exopassalora* herein based on phylogenetic evidence.

***Pseudorepetophragma*** Phookamsak, Bhat and Hongsanan, gen. nov.

*Index Fungorum number*: IF 900624

*Etymology*: The generic epithet “*Pseudorepetophragma*” refers to the genus that is morphologically resembling *Repetophragma*.

Fungus associated with sooty blotch on living leaves of *Zygopetalum mackayi* (*Orchidaceae*). **Sexual morph**: Undetermined. **Asexual morph**: *Colonies* effuse, black, forming a dark mycelial mat. *Mycelium* superficial, composed of septate, branched, dark brown hyphae. *Conidiophores* macronematous, mononematous, simple, erect, septate, straight, or slightly narrow toward the apex, percurrently proliferating, septate, dark brown to brown. *Conidiogenous cells* monoblastic, enteroblastic, integrated, terminal, conspicuously percurrent, with apices remaining wavy or uneven after each conidial secession. *Conidia* acrogenous, solitary, brown, dark brown, cylindrical to obclavate, with truncate base, septate, thick, and smooth-walled. *Conidial secession* schizolytic (adapted from Buyck et al., 2017).

*Type species*: ***Pseudorepetophragma zygopetali*** (O.L. Pereira, Meir. Silva and R.F. Castañeda) Phookamsak, Bhat and Hongsanan

*Notes*: Subramanian (1992) established the genus *Repetophragma* and accommodated some species previously described as *Sporidesmium*. The genus is characterized by macronematous, brown, solitary, septate conidiophores, with monoblastic, enteroblastic, integrated, terminal, percurrently proliferating, and distinctly annellidic conidiogenous cells and acrogenous, solitary, dry, euseptate, conidia with a truncate base (Subramanian, 1992; Castañeda-Ruiz et al., 2011). Castañeda-Ruiz et al. (2011) re-illustrated the genus by providing the synopsis table of morphological features and key to the species of *Repetophragma*. Based on this comprehensive study, Castañeda-Ruiz et al. (2011) introduced a novel species, *R. paracambrense*, and 12 new combinations in the genus. Of these, most species were previously known in *Endophragmiella* and *Sporidesmium*. Considering the species of *Repetophragma*, most of the accepted species have annellidic, percurrent proliferations of the conidiogenous cells bearing euseptate conidia with apically rounded, well-defined, and without rostrate or appendiculate apical cell (Iturriaga et al., 2008; Silvera-Simón et al., 2009). This led to the inclusion of morphologically diverse species in *Repetophragma*. The phylogenetic analyses inferred by Shenoy et al. (2006) and Hernández-Restrepo et al. (2017) also revealed the status of some *Repetophragma* to be polyphyletic. Unfortunately, the phylogenetic affinity of *Repetophragma* is uncertain due to the lack of molecular data for the type species of *Repetophragma*. Furthermore, *R. zygopetali* formed an independent lineage within the *Phaeothecoidiellaceae*. Morphologically, *R. zygopetali* is similar to *R. biseptatum* in having monoblastic, enteroblastic, conspicuously percurrent conidiogenous cells. However, *R. zygopetali* can be distinguished from the rest of *Repetophragma* in having integrated, monoblastic, conspicuously percurrent but irregularly distanced conidiogenous cells with wavy or uneven apices after each conidial secession [Figure 29a in Buyck et al. (2017)] rather than equidistantly laid annellidic conidiogenous cells with even apices after each conidial secession (Subramanian, 1992; Seifert et al., 2011). Based on the morphological differences in the conidiogenous cells and phylogenetic analyses, we introduce the new genus *Pseudorepetophragma* to accommodate *R. zygopetali* as *P. zygopetali* comb. nov.

***Pseudorepetophragma zygopetali*** (O.L. Pereira, Meir. Silva and R.F. Castañeda) Phookamsak, Bhat and Hongsanan, comb. nov.

*Index Fungorum number*: IF 900625

≡ *Repetophragma zygopetali* O.L. Pereira, Meir. Silva and R.F. Castañeda, in Buyck, Duhem, Das, Jayawardena, Niveiro, Pereira, Prasher, Adhikari, Alberto, Bulgakov, Castañeda-Ruíz, Hembrom, Hyde, Lewis, Michlig, Nuytinck, Parihar, Popoff, Ramirez, Da Silva, Verma and Hofstetter, Cryptog. Mycol. 38(1): 135 (2017)

*Detailed description*: See Buyck et al. (2017).

*Notes*: *Pseudorepetophragma zygopetali* was first introduced as *Repetophragma zygopetali* by Buyck et al. (2017) due to its morphological resemblance with *R. dennisii*, but differing in the size of conidiophores and conidia (Castañeda-Ruiz et al., 2011; Buyck et al., 2017). The species was reported as a sooty blotch fungus that occurred on *Zygopetalum mackayi* in Brazil, while other species of *Repetophragma* have been mostly reported as hyperparasites or saprobes (Ellis, 1963; Castañeda-Ruiz et al., 2011; Index Fungorun, 2023). Its life mode is fitted well with the genera in *Phaeothecoidiellaceae*, and this was confirmed by phylogenetic evidence.

***Pseudostomiopeltis*** Phookamsak and Hongsanan, gen. nov.

*Index Fungorum number*: IF 900626

*Etymology*: The generic epithet “*Pseudostomiopeltis*” refers to the genus that resembles *Stomiopeltis*.

*Epiphytic* or *saprobic* on leaves and fruits. **Sexual morph**: *Mycelium* absence. *Ascomata* thyriothecial, black, solitary, gregarious, superficial, rounded, easily removed from the host surface. *Upper wall* composed of a thin layer of neatly arranged dark cells of *textura angularis. Hamathecium* lacking pseudoparaphyses. *Asci* 4-spored, bitunicate, fissitunicate, oblong to subglobose, with a minute pedicel. *Ascospores* uniseriate, hyaline, asymmetric, obovoid to ellipsoid, 1-septate, constricted at the septum, upper cell slightly broader than the lower cell (Adopted from Jayasiri et al., 2019). **Asexual morph**: *Mycelium* absence. *Conidiomata* thyriothecial, superficial, scattered, or in a small group, hemispherical, dimidiate-scutate, uniloculate, with a central, pore-like ostiole. *Upper wall* composed of a thin layer, of brown, radiating cells, with loosely irregular lobed cells at the margin. *Peridium* composed of 1–2 strata of *textura angularis. Conidiophores* reduced to conidiogenous cells. *Conidiogenous cells* arising from the innermost wall cells of the conidiomata, hyaline, enteroblastic, phialidic, lageniform to ampulliform, determinate, discrete, smooth-walled, with minute channel and collarette. *Conidia* solitary, hyaline, ellipsoidal to oblong, slightly truncate at the base, with obtuse apex, aseptate, smooth-walled, occasionally with attached conidiogenous cell. *Sporulation in vitro* forming dense, brown, compact hyphae on OA medium, with cream spore masses. *Conidiophores* hyaline, cylindrical to subcylindrical, septate, branched or unbranched, smooth-walled. *Conidiogenous cells* hyaline, enteroblastic, phialidic, terminal and intercalary, cylindrical to subcylindrical, aseptate, smooth-walled, with minute channel and collarette. *Conidia* solitary, hyaline, subglobose to ellipsoidal to oblong, with obtuse ends, aseptate, smooth-walled.

*Type species*: ***Pseudostomiopeltis xishuangbannaensis*** Phookamsak, Hongsanan, Wanas. and Bhat

*Notes*: Luttrell (1946) re-circumscribed the genus *Stomiopeltis* based on morphological studies and divided the species of *Stomiopeltis* into two groups based on the difference in the types of upper wall cell arrangements. The first group included the type species (*S. aspersa*) that has non-radiating upper wall cells, composed of disorderly arranged, irregularly lobed pseudoparenchymatous cells, while the second group has radiating upper wall cells, somewhat obscured by the curving and twisting of the radiating hyphae and by the irregularly lobed cells, which may be termed as “meandering plectenchyma”. Luttrell (1946) mentioned that the second group should be placed in *Microthyriaceae*. The phylogenetic analyses conducted by Renard et al. (2020) also showed that *Stomiopeltis* is polyphyletic due to *S. betulae* forming a clade within *Microthyriales*, while two *Stomiopeltis*-like species formed a clade with *Tothia fuscella* in *Venturiales*. The present phylogenetic analyses of a concatenated ITS and LSU sequence dataset demonstrated that our new isolate formed a well-resolved subclade with other *Stomiopeltis sensu lato* in *Phaeothecoidiellaceae*, *Mycosphaerellales*. Besides, the type species of *Stomiopeltis*, *S. aspersa*, has not yet been sequenced, and hence the phylogenetic affinity of *Stomiopeltis sensu stricto* is still uncertain.

Zeng et al. (2018) re-examined the holotype of *Stomiopeltis aspersa* and provided an updated morphological description that is characterized by superficial, brown, reticulate hyphae, flattened, circular, brown, thyriothecia with an irregular central ostiole. The upper wall comprises brown, meandrous, compact hyphae, lacking a basal plate. Asci are 8-spored, ellipsoidal, short-pedicellate, with an ocular chamber and ascospores are overlapping 2–3-seriate, hyaline, cylindrical, 1-septate, not constricted at the septum, with the upper cell shorter and broader than the lower cell. Morphologically, our new isolate could not be compared with the type of *S. aspersa* because they form different morphs. However, the new isolate is clearly distinguished from *S. aspersa* by the absence of superficial, brown, reticulate hyphae that penetrate the host and form hemispherical, dimidiate-scutate thyriothecia. Additionally, the upper wall of the thyriothecia radiates, and the cells at the margin are loosely and irregularly lobed, while *S. aspersa* has a non-radiating upper wall composed of sinuous, irregularly lobed cells [Figures 20c, d in Zeng et al. (2018)]. Furthermore, *S. phyllanthi* which formed a clade with our new isolate, is also different from *S. aspersa* in lacking superficial, reticulate hyphae on the host and pseudoparaphyses (Jayasiri et al., 2019). Based on phylogenetic evidence and morphological distinctiveness with the type of *Stomiopeltis*, we introduced the new genus *Pseudostomiopeltis* to accommodate the new species, *Ps. xishuangbannaensis*, while *Stomiopeltis phyllanthi* is also transferred to the new genus as *Pseudostomiopeltis phyllanthi* comb. nov.

***Pseudostomiopeltis phyllanthi*** (Jayasiri, E.B.G. Jones and K.D. Hyde) Phookamsak and Hongsanan, comb. nov.

*Index Fungorum number*: IF 900639

≡ *Stomiopeltis phyllanthi* Jayasiri, E.B.G. Jones and K.D. Hyde, in Jayasiri, Hyde, Jones, McKenzie, Jeewon, Phillips, Bhat, Wanasinghe, Liu, Lu, Kang, Xu and Karunarathna, Mycosphere 10(1): 131 (2019)

*Detailed description*: See Jayasiri et al. (2019).

*Notes*: *Pseudostomiopeltis phyllanthi* was first introduced as *Stomiopeltis phyllanthi* by Jayasiri et al. (2019) which was found as a saprobe on the fruits of *Phyllanthus emblica*. The species formed a sexual morph and is characterized by black, superficial, rounded thyriothecia, with the upper wall neatly lined by dark cells of *textura angularis*, lacking pseudoparaphyses, with 4-spored, fissitunicate, oblong to subglobose asci, and hyaline, obovoid to ellipsoid, 1-septate ascospores (Jayasiri et al., 2019). The present phylogenetic analyses of a concatenated ITS and LSU sequence dataset demonstrated that the species formed a separate branch and is basal to the clade *Pseudostomiopeltis* with significant support (90% ML, 0.98 PP; **Figure 1**). Therefore, we transferred *S. phyllanthi* to *Pseudostomiopeltis* as *Ps. phyllanthi* comb. nov.

**Figure 2:**
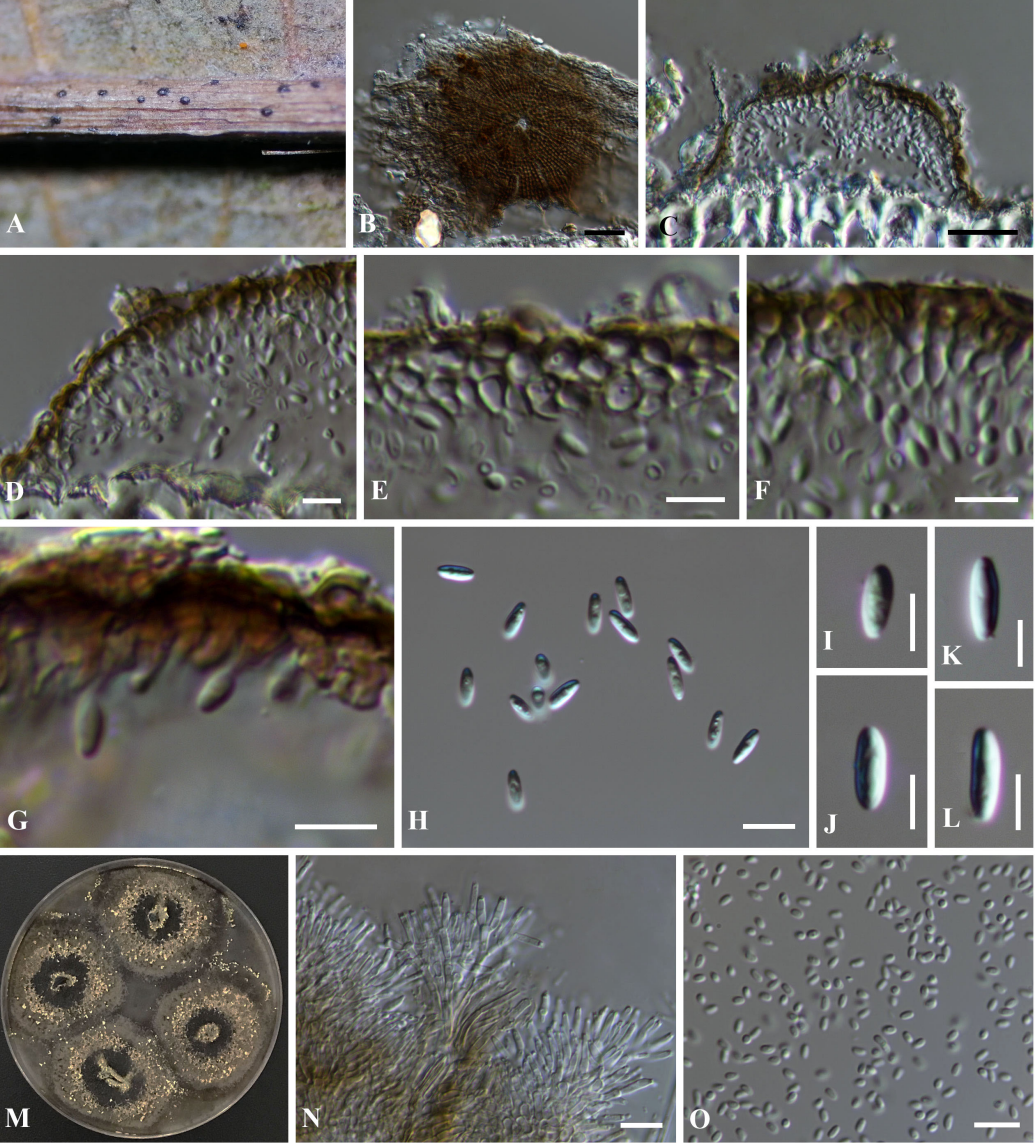
*Pseudostomiopeltis xishuangbannaensis* (KUN-HKAS 129044, holotype). **(A)** The appearance of conidiomata on host substrate. **(B)** Upper view of conidioma. **(C)** Vertical section of conidioma. **(D)** Section through the peridium. **(E–G)** Conidiogenous cells bearing conidia, arising from the inner cavities (note: **G** = stained with Congo red). **(H–L)** Conidia. **(M)** Sporulation on OA with cream conidial masses. **(N)** Conidiophores and conidiogenous cells *in vitro*. **(O)** Conidia sporulated *in vitro*. Scale bars: **(B, C)** = 20 μm, **(D–L, N, O)** = 5 μm.

***Pseudostomiopeltis xishuangbannaensis*** Phookamsak, Hongsanan, Wanas. and Bhat, sp. nov., **Figure 2**

*Index Fungorum number*: IF 900640

*Etymology*: The specific epithet “*xishuangbannaensis*” refers to the locality, Xishuangbanna Dai Autonomous Prefecture, Yunnan, China, where the holotype was collected.

Holotype: KUN-HKAS 129044

*Epiphytic* or *saprobic* on dead leaves of an unidentified dicot, hypophyllous, visible as small, circular, black dots, easily removed from the host surface. **Sexual morph**: Undetermined. **Asexual morph**: *Conidiomata* 85–120 µm high, 70–135 µm, thyriothecial, scattered, or in a small group, hemispherical, dimidiate-scutate, uni-loculate, with a central, pore-like ostiole. *Upper wall* composed of a thin layer, of brown, radiating cells, with loosely irregular lobed cells at the margin. *Peridium* 1.5–4 µm wide, composed of 1–2 strata of *textura angularis. Conidiophores* reduced to the conidiogenous cells. *Conidiogenous cells* 2.5–5 × 1.5–3 µm (*x̄* = 3.9 × 2.3, *n* = 30), arising from the innermost wall cells of the conidiomata, hyaline, enteroblastic, phialidic, lageniform to ampulliform, determinate, discrete, smooth-walled, with minute channel and collarette. *Conidia* 7–9 × 2–4 µm (*x̄* = 8.1 × 3.2, *n* = 30), solitary, hyaline, ellipsoidal to oblong, slightly truncate at the base, with obtuse apex, aseptate, smooth-walled, occasionally with attached conidiogenous cell.

Culture characteristics: Conidia germinated on PDA within 24 h. Colonies on OA reaching 25–28 mm in diam. after two weeks at room temperature (15°C–20°C). Colonies dense, circular, flattened to slightly raised, surface smooth with an entire edge, fairly fluffy to floccose; from above gray at the margin, becoming dark gray toward the center, with cream conidial masses; from below dark gray to black at the margin, white-gray at the middle, dark gray to black at the center, radiating. Sporulation in OA after two weeks, forming dense, brown, compact hyphae on OA medium, with cream spore masses. Mycelium 1.5–3 µm thick, brown, branched, septate, with compact hyphae. Conidiophores 10–30 × 1–3 µm (*x̄* = 13.9 × 2.2, *n* = 30), hyaline, cylindrical to subcylindrical, septate, branched or unbranched, smooth-walled. Conidiogenous cells (5–)7–9 × 1–3 µm (*x̄* = 7.8 × 1.9, *n* = 30), hyaline, enteroblastic, phialidic, terminal, cylindrical to subcylindrical, aseptate, smooth-walled, with minute channel and collarette. Conidia 2.5–4 × 1–2 µm (*x̄* = 3 × 1.7, *n* = 30), solitary, hyaline, subglobose to ellipsoidal to oblong, with obtuse ends, aseptate, smooth-walled.

Material examined: China, Yunnan Province, Xishuangbanna Dai Autonomous Prefecture, Mengla County, Menglun, Xishuangbanna Tropical Botanical Garden, on dead leaves of an unidentified dicot, 11 January 2021, D.N. Wanasinghe, Xh5 (KUN-HKAS 129044, holotype), ex-type living culture, RPC 21-031 = KUNCC.

*Notes*: The NCBI nucleotide BLAST search of ITS sequence indicated that *Pseudostomiopeltis xishuangbannaensis* (RPC 21-031) is similar to *Stomiopeltis* sp. T49A1c with 99.82% similarity (identities = 557/558 with no gap), *Stomiopeltis* sp. T36A1b with 97.32% similarity (identities = 545/560 with 5 gaps), *Stomiopeltis* sp. RS7 with 96.25% similarity (identities = 539/560 with 5 gaps), and *S. phyllanthi* MFLU 18-2115 with 92.36% similarity (identities = 447/484 with 9 gaps). Similarly, the NCBI nucleotide BLAST search of LSU sequence indicated that *Ps. xishuangbannaensis* (RPC 21-031) is similar to Cf. *Stomiopeltis* sp. RS7.2 with 100% similarity (identities = 765/765, with no gap), Cf. *Stomiopeltis* sp. RS7.1 with 99.87% similarity (identities = 764/765, with no gap), and *Stomiopeltis* sp. RS7 with 99.76% similarity (identities = 834/836, with 1 gap), while the LSU of *S. phyllanthi* MFLU 18-2115 is misidentified. The closest hit using the LSU sequence of *S. phyllanthi* is *Meyerozyma guilliermondii* culture CBS:8105 with 100% similarity.

The phylogenetic analyses of a concatenated ITS and LSU sequence dataset (**Figure 1**) revealed that *Pseudostomiopeltis xishuangbannaensis* (RPC 21-031) has a close relationship with *Ps. phyllanthi* MFLU 18-2115 (≡ *Stomiopeltis phyllanthi*). A nucleotide pairwise comparison of ITS sequence indicated that *Ps. xishuangbannaensis* (RPC 21-031) differs from *Ps. phyllanthi* (MFLU 18-2115) in 38/485 bp (7.8%). Morphologically, *Ps. xishuangbannaensis* (RPC 21-031) could not be compared with *Ps. phyllanthi* as they formed different morphs.

## 4 Discussion

In the present study, two new genera, one new species, and three new combinations are described and illustrated based on morphology and phylogeny. The new genus, *Pseudostomiopeltis*, is introduced to accommodate the type species, *Ps. xishuangbannaensis* sp. nov. and *Ps. phyllanthi* com. nov. This genus belongs to *Phaeothecoidiellaceae* (*Mycosphaerellales*). The members of *Pseudostomiopeltis* share certain similar characteristics with *Stomiopeltis* which has been classified as a genus *incertae sedis* in *Capnodiales* by Wijayawardene et al. (2022). *Stomiopeltis* is a polyphyletic genus, and the sequence data of the type species are not available (Renard et al., 2020). It is interesting to note that the morphology of *Stomiopeltis* shows remarkable similarities to the species in *Micropeltidaceae* (*Micropeltidales*, *Lecanoromycetes*). However, molecular analysis has revealed that the majority of *Stomiopeltis* strains are classified in *Phaeothecoidiellaceae* (*Mycosphaerellales*), and most of these strains have not yet been identified at the species level. In the phylogenetic tree constructed using ITS and LSU sequence data (**Figure 1**), our new isolate clustered together with Cf. *Stomiopeltis* sp. RS7.2. Since the strain Cf. *Stomiopeltis* sp. RS7.2 has not been identified as its morphology is not available, we thus establish our strain as a new species. Whereas, *S. phyllanthi* is grouped with the clade of *Pseudostomiopeltis* with significant support (90% ML, 0.98 BYPP; **Figure 1**). Therefore, we transfer *S. phyllanthi* to *Pseudostomiopeltis* based on phylogenetic evidence, although the upper wall structure of *S. phyllanthi* could not be determined in this study.

*Stomiopeltis syzygii* is morphologically similar to *Pseudostomiopeltis xishuangbannaensis* sporulated *in vitro* in having subcylindrical, septate, branched or unbranched conidiophores, terminal and intercalary, phialidic, subcylindrical, hyaline conidiogenous cells, and hyaline, smooth, aseptate conidia (Crous et al., 2019). However, *S. syzygii* has slightly larger [(5–)8–10(–12) × 1.5 µm vs. 2.5–4 × 1–2 µm], subcylindrical conidia, whereas *Ps. xishuangbannaensis* sporulated *in vitro* has subglobose to ellipsoidal or oblong conidia. The phylogenetic analyses demonstrated that *S. syzygii* (CPC 36323, ex-type strain) formed a subclade with *Stomiopeltis* sp. RS1 PEC6a basal to *Pseudostomiopeltis* with low support. Hence, the species is tentatively excluded from *Pseudostomiopeltis* until taxon samplings are increased, providing a better phylogenetic resolution of *Pseudostomiopeltis* with uncertain *Stomiopeltis* spp. within *Phaeothecoidiellaceae*. It is notable that *S. betulae* clustered within *Microthyriales*. However, the phylogenetic placements of *Stomiopeltis* and its relationships with other genera remain unclear. Further sequence data and morphological studies are needed to confirm the placement of this genus.

Besides the phylogenetic investigation of *Stomiopeltis sensu lato* in *Phaeothecoidiellaceae*, *Repetophragma zygopetali* formed an independent lineage within *Phaeothecoidiellaceae* in the present study. Buyck et al. (2017) treated *R. zygopetali* in *Micropeltidaceae* (*Micropeltidales*, *Lecanoromycetes*) based on phylogenetic evidence that *R. zygopetali* formed a basal clade with *Sporidesmajora pennsylvaniensis* (CPC 16112*)*, *Houjia pomigena* (CPC 16109), and *H. yanglingensis* (CPC 16110, CPC 16111, CPC 16113, and CPC 16114) in their analysis. Unfortunately, most *Repetophragma* species were identified based solely on morphological characteristics that distinguished them from *Sporidesmium* in having conidiophores with terminal annellations, suggestive of repeated percurrent proliferative conidiogenous cells (Subramanian, 1992). Whereas *Sporidesmium* species have non-hyphopodiate mycelium, simple non-proliferating conidiophores, or with irregularly distanced percurrent proliferations (Subramanian, 1992). Presently, *Sporidesmium* was accommodated in its own family (*Sporidesmiaceae*, *Sporidesmiales*, *Sordariomycetes*), whereas the phylogenetic affinity of *Repetophragma sensu stricto* is uncertain. Based solely on morphology, the taxonomy of *Repetophragma* remains ambiguous. Molecular data of species in *Repetophragma* is urgently needed to verify their congeneric status within *Repetophragma* and also clarify the phylogenetic affinity of *Repetophragma*.

*Exopassalora* was established to accommodate *Passalora zambiae* (Crous et al., 2004). The species was isolated from leaf spots of *Eucalyptus globulus* in Zambia. Crous et al. (2004) classified the species into *Passalora* due to its being phylogenetically distinct from other *Mycosphaerella* spp. known from *Eucalyptus*. Later, Videira et al. (2017) introduced many novel genera to accommodate *Passalora sensu lato*, including *Exopassalora*, based on multigene phylogenetic evidence. The sexual morph of *E. zambiae* is known only for its asci and ascospores, prepared onto the slide (Crous et al., 2004). In the present phylogenetic analyses, *Stomiopeltis sinensis* formed a separate branch basal to *Exopassalora*. Hence, the species is transferred to *Exopassalora,* as *E. sinensis* comb. nov., based on phylogenetic evidence. Morphologically, *E. sinensis* could be only compared with *E. zambiae* in ascospore characters that are similar in having hyaline, 1-septate ascospores (Crous et al., 2004; Jayasiri et al., 2019). Moreover, *E. zambiae* was found on leaf spots of *Eucalyptus globulus*, while *Stomiopeltis* spp. clustered in the subclade of *Exopassalora* were found as sooty blotch and flyspeck fungi on apples and pears (Batzer, 2005; Ismail et al., 2016). In contrast, *E. sinensis* was isolated from decaying fruit pericarp of *Harpephyllum* sp. (wild plum). It is presumable that the species may occur on fresh fruits of *Harpephyllum* as parasites and continue living on dead fruits as a saprobe, similar to *Pseudostomiopeltis xishuangbannaensis*. However, the change in life mode may need further study for a better understanding of the pathogenic capabilities of these species.

Most genera of *Phaeothecoidiellaceae* were known as pathogenic fungi causing sooty blotch and flyspeck or leaf spot diseases on various hosts worldwide (Crous et al., 2004; Batzer, 2005; Yang et al., 2010; Ismail et al., 2016; Buyck et al., 2017; Hongsanan et al., 2017; Zeng et al., 2018; Crous et al., 2019; Hongsanan et al., 2020b; Crous et al., 2021). Species of *Chaetothyrina*, *Houjia*, *Phaeothecoidiella*, *Sporidesmajora*, *Stomiopeltis*-like spp., and *Translucidithyrium* have been reported as sooty blotch and flyspeck fungi, mainly occurring on apples and pears (Batzer, 2005; Yang et al., 2010; Ismail et al., 2016; Hongsanan et al., 2017; Zeng et al., 2018; Hongsanan et al., 2020b; Li et al., 2020), while species of *Exopassalora* and *Nowamyces* have been reported as fungi associated with leaf spot diseases (Crous et al., 2004; Crous et al., 2019). *Neochaetothyrina*, on the other hand, has been reported as a saprobe on *Syzygium* (Crous et al., 2021). In the present study, *Pseudostomiopeltis phyllanthi* and *Ps*. *xishuangbannaensis* were found as saprobes on fruits and leaves of dicots. However, *Stomiopeltis*-like spp. clustered with *Pseudostomiopeltis* were reported as sooty blotch and flyspeck fungi on apples (Mayfield et al., 2013). This leads to questioning that *Pseudostomiopeltis* may also be capable of causing sooty blotch and flyspeck disease.

Yunnan is a region known for its high biodiversity, serving as a critical habitat for numerous species. However, this region is under threat from habitat loss, climate change, and various human activities. Among these factors, climate change is a major threat to the biodiversity in Yunnan. As global temperatures continue to rise, the region may experience changes in precipitation patterns and temperature regimes that could potentially impact the distribution and survival of numerous species, including fungi. Despite these challenges, there are also opportunities for biodiversity conservation in Yunnan. Scientific research and monitoring play crucial roles in providing valuable information for the biodiversity conservation efforts in the region. The discovery of novel taxa highlights the rich fungal diversity in Yunnan and contributes to our understanding of the ecological roles of fungi in forest ecosystems. The description and documentation of these new taxa not only provide important information on fungi useful in future research but also enhance our knowledge of conservation efforts in the region. By expanding our understanding of the biodiversity of Yunnan, we can better protect and manage its unique ecosystems and ensure the long-term survival of its species.

## Data availability statement

The data presented in the study are deposited in the GenBank repository, accession numbers OR233594 (LSU) and OR233596 (ITS).

## Author contributions

SH and RP: conceptualization. SH and RP: data curation. SH and DS: formal analysis. SL, JX, IP, and NX: funding acquisition. SH, RP, DB, DW, NS, and DS: investigation. SH, RP, and DS: methodology. SH and NS: project administration. SL, JX, IP, and NX: supervision. SH, RP, DW, NS, and DS: writing—original draft. SH, RP, DB, DW, NS, IP, and DS: writing—review and editing. All authors contributed to the article and approved the submitted version.
